# Heuristic Routing Algorithms for Time-Sensitive Networks in Smart Factories

**DOI:** 10.3390/s22114153

**Published:** 2022-05-30

**Authors:** Yue Li, Zhenyu Yin, Yue Ma, Fulong Xu, Haoyu Yu, Guangjie Han, Yuanguo Bi

**Affiliations:** 1 School of Computer Science and Technology, University of Chinese Academy of Sciences, Beijing 100049, China; liyue161@mails.ucas.ac.cn (Y.L.); mayue@sict.ac.cn (Y.M.); xufulong16@mails.ucas.ac.cn (F.X.); yuhaoyu@sict.ac.cn (H.Y.); 2 Shenyang Institute of Computing Technology, Chinese Academy of Sciences, Shenyang 110168, China; 3 Liaoning Key Laboratory of Domestic Industrial Control Platform Technology on Basic Hardware and Software, Shenyang 110168, China; 4 College of Internet of Things Engineering, Hohai University, Changzhou 213022, China; hanguangjie@gmail.com; 5 Changzhou Key Laboratory of Internet of Things Technology for Intelligent River and Lake, Changzhou 213022, China; 6 School of Computer Science and Engineering, Northeastern University, Shenyang 110167, China; biyuanguo@mail.neu.edu.cn; 7 Engineering Research Center of Security Technology of Complex Network System, Ministry of Education, Shenyang 110167, China

**Keywords:** time-sensitive network, smart factory, industrial internet, routing, heuristic algorithm

## Abstract

Over recent years, traditional manufacturing factories have been accelerating their transformation and upgrade toward smart factories, which are an important concept within Industry 4.0. As a key communication technology in the industrial internet architecture, time-sensitive networks (TSNs) can break through communication barriers between subsystems within smart factories and form a common network for various network flows. Traditional routing algorithms are not applicable for this novel type of network, as they cause unnecessary congestion and latency. Therefore, this study examined the classification of TSN flows in smart factories, converted the routing problem into two graphical problems, and proposed two heuristic optimization algorithms, namely GATTRP and AACO, to find the optimal solution. The experiments showed that the algorithms proposed in this paper could provide a more reasonable routing arrangement for various TSN flows with different time sensitivities. The algorithms could effectively reduce the overall delay by up to 74% and 41%, respectively, with promising operating performances.

## 1. Introduction

With the rapid development of the industrial internet, real-time communication technologies with deterministic low latency have become a critical requirement in many industrial sectors. For example, most industrial automation networks require end-to-end latency to be strictly controlled at no more than 1 millisecond [1]. In addition to the latency requirements, most application scenarios also have diverse demands on transmission jitter, packet loss rate, etc., while traditional Ethernet communication can only provide best-effort and soft real-time transmission services. In response to the growing demand for industrial real-time communication, industrial enterprises all over the world have developed various industrial control network protocols based on standard Ethernet communication, such as Real-Time TTEthernet, EtherCAT, PROFINET, SERCOIII, etc. These deterministic industrial networks connect manufacturing equipment and controllers, constituting operation technology (OT) networks [2] that are now widely used.

However, incompatible network protocols lead to problems such as incompatible applications, a lack of interoperability, difficulty in portability, and expensive costs for development, deployment, and maintenance. With the relentless efforts of the AVnu Industry Alliance and the IEEE TSN Working Group (formerly the AVB Working Group), TSNs [3] have emerged as a brand new industrial communication technology that are now being actively promoted by industrial communities. TSNs allow both periodic and non-periodic data to be transmitted within the same network, giving standard Ethernet communication the ability for deterministic transmission. TSNs are constructed on the standard 802.1 Ethernet protocol stacks, which naturally have the advantage of interconnection and can achieve open Layer 2 Forwarding while ensuring deterministic latency bounds and bandwidth guarantees [4]. Therefore, TSNs are able to interconnect mutually isolated information technology (IT) networks and OT networks to achieve the co-networked converged transmission of multiple data flows with varying time sensitivities [5]. In recent years, TSNs have received continuous attention from both academia and industry and have been identified as a key technology for the next generation of industrial communication systems [6,7,8]. With this continuous attention and the efforts of standardization organizations, including the IEEE and IEC, a series of amendments and standards to improve TSN protocols [9,10,11] have been released.

The continuous improvement of TSN-related standardization has become a research trend in the building of a comprehensive industrial internet communication system that is based on a TSN with deep integration of IT and OT [12]. Among many application directions, smart factories, a key component of Industry 4.0, comprise an important application scenario for TSN communication technology. A smart factory is a comprehensive production system with multiple intelligent subsystems at different levels [13], with each subsystem having different needs for industrial communication. While ensuring real-time control data communication for smart factories, only TSNs can realize barrier-free communication with other the subsystems mentioned above within the smart factory architecture [14,15]. For TSNs in smart factories, a novel routing mechanism needs to be established that considers their unique characteristics.

Currently, research on TSNs is still in the development stage and existing data flow route planning mechanisms are relatively simple. In our previous studies [16,17,18], we contributed to the traffic shaping mechanisms along with real-time secure communication methods for joint OPC UA–TSN IoT-based intelligent industrial production lines; however, the depth of discussion around routing for TSNs has much room for improvement. In both mainstream studies and our previous studies, the network topologies were not complicated, routes of all kinds of traffic were determined using spanning tree protocols and shortest path routing algorithms, and port queuing and time slot allocation in frame transmission were performed. However, the composite routing problem for multiple flows within large-scale networks is difficult to solve in polynomial time, so online routing algorithms can hardly meet the growing needs of TSNs [19]. Researchers have turned to developing offline routing methods, which aim to generate a reasonable network routing list in an offline manner through exploiting the characteristics of TSN deterministic communication. During actual communication, data flows are scheduled according to the preset routing list. Compared to online algorithms, this approach avoids the strict computing time limits and can perform more iterations to obtain a better approximate optimal solution. Therefore, we propose an offline TSN routing method that covers both TT flow and non-TT flow routing problems, based on heuristic algorithms. The main contributions of our study are as follows:

1. We proposed an improved genetic algorithm to solve the TT flow routing problem (GATTRP). We modeled the TT flow routing problem in the TSN systems of smart factories and transformed the problem into a multiple traveling salesmen problem (MTSP) to solve. Based on the existing genetic algorithms, we optimized the design of the genetic evolution operators and algorithm processes and finally, formed an improved genetic algorithm with faster convergence speed and better results;
2. We proposed an adversarial ant colony optimization (AACO) algorithm to solve the non-TT flow routing problem. We modeled the non-TT flow routing problem in the TSN systems of smart factories and transformed the problem into a load balancing pathfinding problem for multiple priority flows within a directed graph. Based on existing ACO algorithms, we designed a novel pheromone update rule to balance the impacts of higher priority tasks and path length on pathfinding. The algorithm could effectively equalize the non-TT network load and reduce the network latency;
3. We established a simulation experiment platform for the smart factory TSN communication system and evaluated the performance of the proposed algorithm through experiments. The results showed that both algorithms produced a certain improvement in the corresponding evaluation indicators, which matched our expectations.

The remainder of this paper is organized as follows. In Section 2, we list the results of our survey on related works. In Section 3, we parse and mathematically model the actual problems. In Section 4, we propose the TT routing algorithm, named GATTRP. In Section 5, we propose the non-TT routing algorithm, named AACO. In Section 6, we establish an experimental environment and discuss performance evaluation. Finally, we conclude this paper in Section 7.

## 2. Related Works

Our study combined several fields, such as time-sensitive network routing, task/volume-balanced MTSPs, load balancing routing, etc. We reviewed many studies from the related fields, which are listed below.

### 2.1. Time-Sensitive Network Routing

TSNs are composed of series of IEEE technical standards, including precise time synchronization, network traffic shaping, network configuration, and other aspects. The IEEE TSN Standardization Working Group issued a standard amendment [20] to address the problem of path controlling and proposed shortest path bridging (SPB) as the basis for establishing network bridging in Chapter 12. Based on this, a management information base (MIB) was specified in Chapter 17, which formed the IEEE 8021-SPB-MIB standardization framework. Furthermore, the amendment proposed a path control and reservation (PCR) method in Chapter 45, which was based on the use of SPB and spanning tree protocols to achieve the path control and traffic reservation of TSN flows. Following on from IEEE standards, Schweissguth et al. and Falk et al. [21,22] proposed solutions for the joint traffic scheduling and route planning problem, both of which were based on integer linear programming (ILP). Schweissguth et al. [21] used two performance metrics (i.e., end-to-end delay and scheduling capability) to evaluate their experimental results for different traffic patterns and network topologies. Falk et al. [22] adopted an ILP solver for instances with large parameter variations and evaluated the performance of their algorithm based on the solution time. Mahfouzi et al. [23] proposed an iterative algorithm for joint routing and scheduling based on SMT, but the performance of their algorithm was too sensitive to the degree of transmission path conflicts between flows, which led to an unsatisfactory success rate. Nayak et al. [24,25] proposed the concept of a time-sensitive software-defined network (TSSDN), which forms a logically centralized control plane of SDN to compute global routing schemes. The above studies provided feasible routing mechanisms for TT traffic, but it is difficult to meet the requirements of the solution time for large-scale routing scenarios and no relevant studies have been found that route for non-TT traffic. Therefore, the existing methods can hardly cope with the routing challenges brought by future large-scale TSN communication systems.

### 2.2. Heuristic Optimization Algorithms

Heuristic algorithms are intuitively or empirically constructed algorithms that search for a feasible solution to each instance of an optimization problem at a limited cost (in terms of computational time and space). Since heuristic algorithms can usually find promising solutions in a reasonable amount of time when dealing with many practical NP-hard problems, they have become a research hotspot in recent years. Inspired by various phenomena, such as animal behavior and natural laws, researchers have proposed many novel and effective optimization algorithms and have proven their value in practical applications. For example, based on the gravitational search algorithm (GSA), which was inspired by the law of gravity and interactions between mass entities, Precup et al. [26] proposed the tuning of a class of fuzzy control systems to obtain a reduced sensitivity. Li et al. [27] proposed an effective rule classifying method, namely the heuristic algorithm to reduce memory demand (HARD), for heterogeneous bit-split string matching architectures. Based on the gray wolf optimization (GWO) algorithm, which was inspired by the action of a gray wolf preying on its prey, Zamfirache et al. [28] proposed an RL-based control approach to train neural networks. Pozna et al. [29] proposed a hybrid metaheuristic optimization algorithm called the particle filter–particle swarm optimization (PF–PSO) algorithm, which can effectively optimize the position control of a family of integral-type servo systems. The above works have been proven to be successful in various applications and thus, are valuable for the further improvement of heuristic algorithms. In order to solve the multi-objective task scheduling problem of intelligent production lines, we proposed a hybrid algorithm called the improved hybrid monarch butterfly optimization and improved ant colony optimization algorithm (HMA) [30] to combine the advantages of cloud computing and fog computing. Based on our previous research, we started trying to solve the routing problems in TSN transmission using heuristic algorithms.

### 2.3. Task/Volume-Balanced MTSPs

The traveling salesman problem (TSP) is a typical NP-hard combinatorial optimization problem, which comprises finding the best traversal route at the lowest cost (time, distance, etc.) through a given number of cities, in which all cities are visited only once by a single traveler, except for the starting city [31]. The MTSP, on the other hand, comprises *M* travelers visiting a portion of cities separately and each city (except the starting city) is only visited by any traveler once and, eventually, finding the minimum cost to finish traversing all of the cities [32]. When M=1, MTSP is transformed into classical TSP, so TSP is a special case of MTSP [33].

The genetic algorithm (GA) has definite advantages for solving the task-balanced MTSP problem. Carter et al. [34] proposed a two-stage chromosome encoding method based on classical GA and designed corresponding genetic operators to solve the MTSP both in terms of the shortest total distance and the shortest "longest distance", which could effectively reduce the solution space and eliminate redundant solutions. Zhou et al. [35] successfully proved the advantages of the improved uniparental GA for solving the MTSP, as well as proposing three algorithms to solve the MTSP with multiple starting points and closed loops. Lu et al. [36] combined the K-means clustering algorithm and the GA to solve the multi-objective MTSP, which avoided travelers crossing paths and also reduced computing time. However, the correctness of the results and the convergence performance of the above algorithms still need to be improved as retaining good individuals while maintaining the diversity of the population within the GA for the MTSP remains a challenging problem.

### 2.4. Load Balancing Routing Assignment Problem

When routing in large-scale network systems, the load balancing problem needs to be fully considered to avoid partial network congestion. To solve the load balancing routing problem, some existing clustering protocols for wireless sensor networks (WSNs) have appreciable reference value. The LEACH (low-energy adaptive clustering hierarchy) algorithm [37] was the first proposed hierarchical routing algorithm, whose core idea is to divide the network nodes into clusters and randomly select nodes in turn to be the cluster head nodes. The other nodes forward their collected data to the cluster head node and, eventually, the cluster head node consolidates the data and forward them to the sink node. Younis and Fahmy [38] proposed a HEED clustering approach. Its major difference from the basic LEACH protocol is that HEED uses a multi-hop method to communicate with the sink node, while LEACH uses a single-hop method. Inspired by these two important clustering protocols, Tarhani et al. [39] proposed SEECH, Bhushan et al. [40] proposed FLEAC, and Sert et al. [41] proposed MOFCA, forming a rich variety of clustering routing methods for WSNs that have a better performance.

There are researchers continuously trying to apply ant colony optimization (ACO) algorithms to solving the load balancing routing assignment problem. ACO is an intelligent optimization algorithm that optimizes practical problems by imitating the foraging behavior of ants in nature, which was first proposed by Italian scholar Marco Dorigo in the 1990s [42]. Ramamoorthy et al. [43] proposed an enhanced hybrid ant colony optimization routing protocol (EHACORP) to improve the efficiency of the routing process using the shortest path. Belgaum et al. [44] explored two artificial intelligence optimization techniques, including ACO and PSO, for load balancing in SDN. Govardhan and Srinivasan [45] proposed a modified evolutionary computing-driven dynamic load balancing model, named intrinsically modified ant colony system (IMACS), for mega-cloud infrastructures. The above algorithms have different degrees of optimization for the route assignment problem of load balancing. However, to the best of our knowledge, there is still a lack of well-performing load balancing route assignment methods for TSN multi-priority scheduling characteristics.

## 3. Problem Modeling

**Figure 1:**
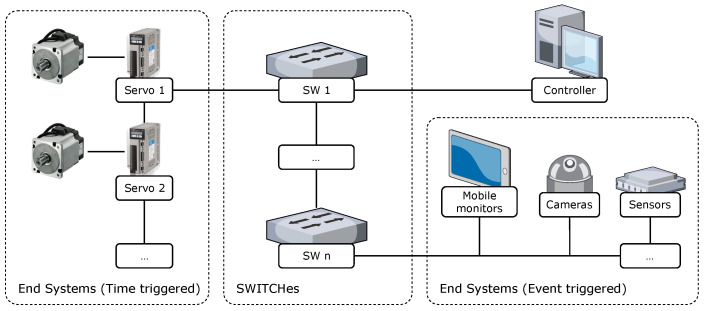
The architecture of a TSN in a smart factory and the composition of the TSN nodes.

To study the TSN routing problem within a complex network topology, the network was abstracted as a directed graph G(V,E). The nodes in this network included two main types: switch (SW) nodes and end system (ES) nodes, as shown in Figure 1. All of the nodes formed a node set *V* and each SW had multiple incoming and outgoing ports, which were responsible for forwarding the data frames received from the incoming ports to the corresponding outgoing ports, according to the routing list. The ESs could be hard real-time control units, such as servo drivers, or soft real-time or non-real-time units, such as sensors, cameras, mobile operating terminals, etc. E⊆V×V was the set of edges, where each element represents a unidirectional link from one node to another. The TSN connections supported full-duplex, so the physical links between node $v_{i}$ and $v_{j}$ corresponded to two directed edges [$v_{i},v_{j}$] and [$v_{j},v_{i}$] in the model. Each link [$v_{i},v_{j}$] was defined by a triplet $<b_{ij},c_{ij},q_{ij}>$, which denoted the bandwidth capacity, propagation delay, and queuing delay of that link, respectively.

Based on the common TSN traffic classification method [7,46,47], we classified the industrial data transmitted by TSN industrial communication systems in smart factories into three main types. For high-precision servo motors in key manufacturing equipment, such as computer numerical control (CNC) machine tools and six-axis robotic arms, the master unit needs to periodically send control data, along with time-synchronized data, as defined in [9,48], which are collectively called time-triggered (TT) data. Meanwhile, some upper-level industrial applications that rely on computer vision, such as object recognition inspection systems for workpiece shapes, require access to audio and video surveillance streams throughout the manufacturing process. These data, collectively called audio/video bridging (AVB) data, are less time-sensitive than TT data and, therefore, need to be scheduled for transmission after the highest priority TT data. In addition, TSNs also provide non-real-time transmission services for upper industrial integrated management systems, such as ERP and MES, in smart factories. These communication systems are not considered within the QoS of real-time industrial networks, collectively called best-effort (BE) communication. A summary of the above three types of data is depicted in Table 1.

AVB and BE data cannot be transmitted in hard real-time, as with TT data, so we defined them as the same type of data, i.e., non-TT data, in our study. In the following sections, we define the two different routing problems, TT and non-TT, and propose two different optimization algorithms to solve these routing problems.

## 4. Improved Genetic Algorithm to Solve TT Flow Routing Problem

**Figure 2:**
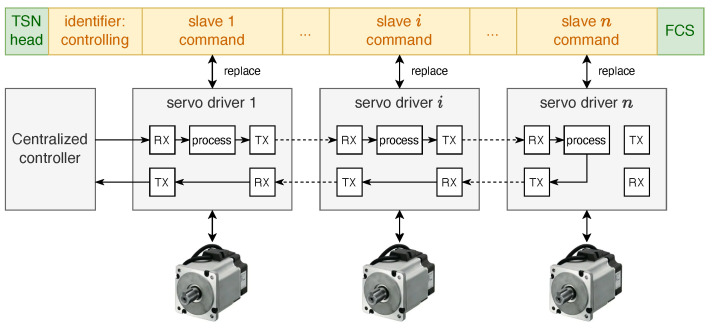
Aggregated forwarding frame-based TT communication.

When the controller conducts time-triggered communication with servo motors within TT subnetworks, the communication method is the master–slave method, in which the controller acts as the master station to send control-type frames to each slave station and slave stations return the processed frames to the master station. In the IT–OT converged industrial control network considered in this paper, the technical idea of aggregated forwarding frames was adopted, in which the whole subsystem has only one frame that runs in a loop. For the master, all devices with I/O information are considered as “logical” devices and the address of the field device corresponds to the physical location in the control frame, according to the protocol. When a message passes through a slave device, the slave only needs to read the command data from the corresponding mapped address and simultaneously resend the feedback data to the same place, so the effective utilization of the message can reach up to more than 90%. The frame format for the control data and communication mechanism design are shown in Figure 2.

**Figure 3:**
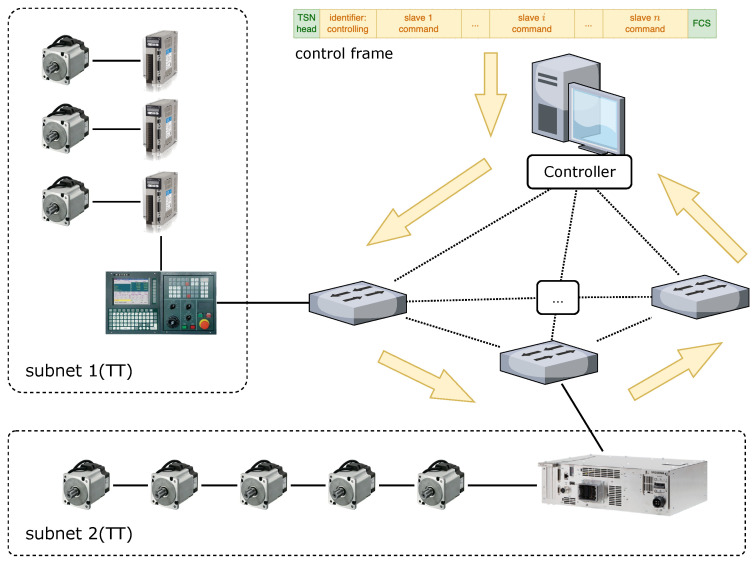
Schematic of TT control flow network traversal.

In industrial control networks, the number of slaves that need to be traversed in single time cycle increases as the network size continues to grow and the time cycle that is required to traverse the nodes increases linearly. In this case, we considered a TT control network based on the idea of distributed control, as depicted in Figure 3. In smart factories, due to the high degree of integration of IT and OT networks, the central control server can combine the requirements from upper-level industrial applications and the cloud platform to simultaneously control CNC machine tools, industrial robots, and other key manufacturing equipment on multiple smart production lines in real time. Each CNC system, six-axis robot arm, etc., forms a set of subnetworks with its own hard real-time requirements within the system. The central control server accesses the submasters of each subnetwork sequentially via SWs, controls them with TT frames, and collects feedback. By optimizing the traversal route, we can reduce the traversal period as much as possible, so that the access time interval from the central control server to each subnetwork can be significantly reduced. The more frequent the access to the manufacturing equipment in the same time slice, the faster the speed of command response and the higher the manufacturing accuracy and flexibility of the smart production line. Therefore, the optimization objective for TT flows in this paper was to reduce the traversal cycle from the central control server to each real-time subnetwork.

### 4.1. Definition of the Optimization Problem in TT Routing

Based on the distributed traversal approach for TT communication that was proposed above, the TT routing problem in TSNs could be transferred into the following: the central control node $v_{1}$ was directly connected to *m* SW nodes and periodically sent *m* control-type data frames (noted as $a_{1},a_{2},\cdots ,a_{m}$) to *n* destination subnetworks at the same time. After each subcontrol system finished receiving and processing, it overwrote the control data field at the corresponding position within the frame with feedback data containing operating status and then forwarded it downward. Finally, all frames converged at $v_{1}$ after the traversal. Every subnetwork that was connected to a SW node could be merged into the model. Considered as a single node, the processing delay of the node equaled the total traversal time inside the node. We let the number of ES nodes inside subnetwork $v_{i}$ be $o_{i}$ and the time granularity of traversing a single ES node be δ, then the total time delay $d_{ij}$ between node $v_{i}$ and $v_{j}$ included the propagation delay between the two nodes, the queuing delay, and the processing delay required to traverse within the $v_{i}$ node:

(1) $$d_{ij}=c_{ij}+q_{ij}+\delta \cdot o_{i}.$$

The minimum time that was required to complete a cycle equaled the time required for the longest of all sent frames to complete forwarding and return to the source node, i.e., the minimum value of cycle *T* was:

(2) $$T=\min\left(\max\sum_{i=1}^{n}\sum_{j=1}^{n}d_{ij}\cdot \rho_{ij}^{k}\right),\;\forall k\in \left\{1,2,\cdots ,m\right\},$$

where $\rho_{ij}^{k}$ is the transmission direction of the TT frame between nodes $v_{i}$ and $v_{j}$, which is defined as follows:

(3) $$\rho_{ij}^{k}=\left\{\begin{array}{cc} 1, & \mathrm{TT}\;\mathrm{frame}\;k\;\mathrm{goes}\;\mathrm{from}\;i\;\mathrm{to}\;j\\ 0, & \mathrm{TT}\;\mathrm{frame}\;k\;\mathrm{goes}\;\mathrm{from}\;j\;\mathrm{to}\;i \end{array}\right..$$

For each *k*th TT frame, we planned a loop route starting from $v_{1}$. Assuming the route was $v_{1}\to v_{2}\to v_{3}\to \cdots \to v_{6}\to v_{1}$, there had to be $\rho_{1,2}^{k}=\rho_{2,3}^{k}=\cdots =\rho_{6,1}^{k}=1$, while any other $\rho_{ij}^{k}=0$. Then, we obtained the 1/m part of the solution: route $\omega_{k}=\left(v_{2},v_{3},\cdots ,v_{6}\right)$.

In addition, we defined $y_{i}^{k}$ to mark whether the *k*th TT frame had visited node $v_{i}$ as follows:

(4) $$y_{i}^{k}=\left\{\begin{array}{cc} 1, & \mathrm{TT}\;\mathrm{frame}\;\mathrm{transmitted}\;\mathrm{through}\;\mathrm{node}\;v_{i}\;\mathrm{once}\\ 0, & \mathrm{others} \end{array}\right..$$

Each node $v_{i}$ was marked one time per TT frame arrival; therefore, for every node $v_{i}$, there was:

(5) $$y_{i}^{k}=\sum_{j=0}^{n}\rho_{ji}^{k},\;\forall i\in \left\{1,2,\cdots ,n\right\},\;k\in \left\{1,2,\cdots ,m\right\}.$$

By treating the node $v_{i}$ as a city, the links between nodes $e_{ij}$ as inter-city paths, and the *m* TT frames transmitted in parallel as *m* traveling salesmen, we could further transform the TT routing problem into an MTSP problem.

### 4.2. Optimization Goal and Constraints of TT Routing

Through the description of the above formula, our goal formula became clear: comparing the completion times of all *m* salesmen to find the longest route. Our goal was to minimize the completion time of the salesman who traveled the longest route by adjusting the routing plans for all of the salesmen, namely:

(6) $$\begin{array}{ccc} \; & \; & Minimize\{T\},\\ s.t. & (c1): & \sum_{k=1}^{m}y_{1}^{k}=m,\\ \; & (c2): & \sum_{k=1}^{m}y_{i}^{k}=1,\;\forall i\in \left\{2,3,\cdots ,n\right\},\\ \; & (c3): & \sum_{i=1}^{n}y_{i}^{k}\geq 2,\;\forall k\in \left\{1,2,\cdots ,m\right\},\\ \; & (c4): & T\leq MaxTime. \end{array}$$

In Equation (6), (c1)–(c4) are the constraints that needed to be obeyed by any solution. Constraint (c1) ensured that *m* TT frames all returned to the central control node within a single common traversal cycle. Constraint (c2) ensured that every node $v_{i}$, except for $v_{1}$, was visited only one time because a node being accessed two or more times within one cycle would break the transmission periodicity and thus, cause chaos in the whole network. Constraint (c3) ensured that each route included at least one node in addition to the central control node. Constraint (c4) ensured that the final result *T* was less than the preset threshold MaxTime, otherwise its real-time performance could not be guaranteed.

### 4.3. Description of GATTRP

In this section, we propose an improved GA named GATTRP. Through GATTRP, we could encode the routes of the TT flows and seek the optimal solution for this problem in a genetic evolutionary way. In the following content, we describe how the improved GA works, starting with the rules by which the chromosomes are encoded.

#### 4.3.1. Chromosome Encoding Rules

In order to reduce the search space and eliminate redundant solutions, this paper adopted a two-segment chromosome encoding method. The first segment represented the order of salesmen traversing each node and the second segment represented the breakpoints between each salesman. When there were *n* nodes, the first node $v_{1}$ was fixed as the starting and destination node for salesmen to traverse; the other n−1 nodes were randomly arranged to be visited by *m* salesmen. The fixed starting node was not encoded in the chromosome and the length of the first part of the chromosome was n−1, indicating the random arrangement of n−1 nodes. The length of the second part was m−1, indicating that when the nodes needed to be divided into *m* salesmen’s paths, m−1 breakpoints were needed. The breakpoints in the second part were stored in increasing order.

**Figure 4:**
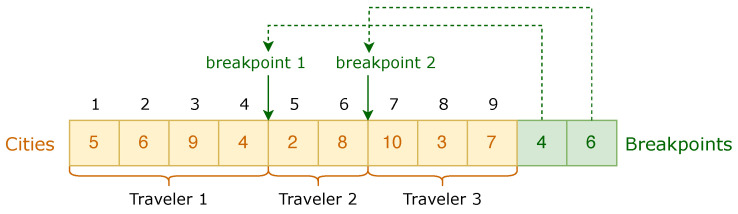
Encoding method for an example chromosome.

In the following example, we made n=10,m=3, and the number of the fixed starting node be 1. We let the randomly generated breakpoints be 4 and 6. Then, we could encode the chromosome as is depicted in Figure 4. The traversal route of the first salesman was 1→5→6→9→4→1, the traversal route of the second salesman was 1→2→8→1, and the traversal route of the third salesman was 1→10→3→7→1.

**Figure 5:**
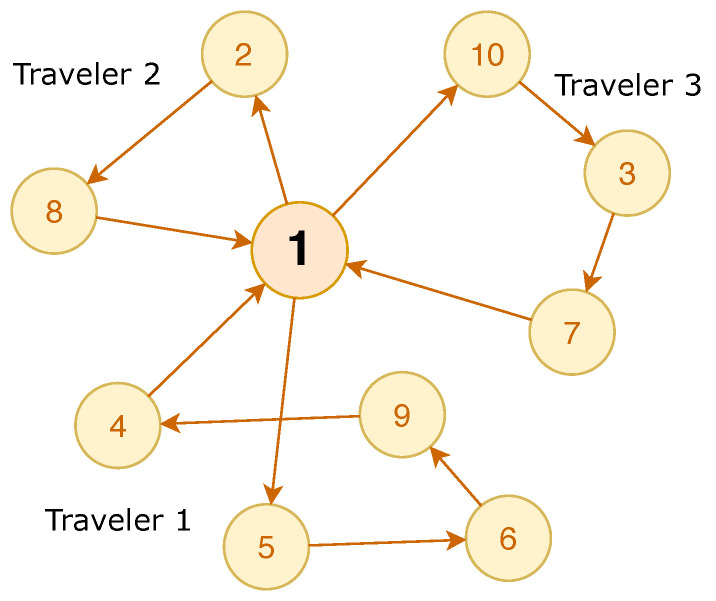
The traversal route of the chromosome in Figure 4.

The above chromosome corresponds to the route of TT flows traversing in the actual network as depicted in Figure 5.

#### 4.3.2. Population Initialization

We initialized a population with an initial chromosome number of $U_{0}$ and we set the maximum population size allowed throughout the iteration as $U_{max}$ and the maximum number of iterations as $I_{max}$. The gene segments of $U_{0}$ chromosomes were randomly initialized and the fitness of individuals on each chromosome was calculated.

#### 4.3.3. Genetic Evolution Operator Design

We combined simple operators, such as the *flip, slide, swap* mutation, to design relatively complex operators, which improved the diversity of the mutation process, accelerated the evolution process, and, eventually, enhanced the efficiency of the GA.

**Figure 6:**
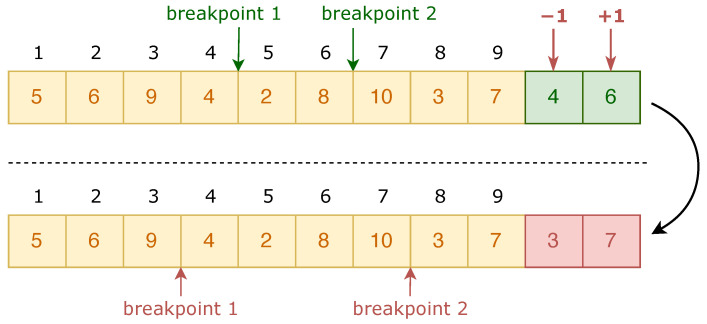
Example process of drift mutation.

In existing mutation operators, the route length of each traveling salesman is constant, which is not enough to effectively improve the population diversity and it is detrimental to locally search for new possible solutions. Therefore, for the second segment of the chromosome, the operation of +*n*, -*n* or +0 was randomly applied to the breakpoint gene segments on the premise that the breakpoints were not equal to each other and were arranged in ascending order. The effect of the above operations was a change in the length of the salesmen’s routes. The mutation process is depicted in Figure 6.

#### 4.3.4. Offspring Breeding

The optimization goal of the improved GA proposed in this section was to find the traversal route with the earliest "latest arrival" time of multiple traveling salesmen. Therefore, Equation (2) was determined as the fitness function for this section. We defined fitness $fit_{i}$ as the evaluation criteria when breeding the *i*th chromosome, where $fit_{i}=1/\text{min}\left(T\right)$. After generating the primary population according to the above chromosome encoding method, chromosomes with better fitness were selected to breed more offspring, so that they could keep their genetic advantages in the evolutionary iteration process. The specific number of reproductions of each chromosome was:

(7) $$O_{i}=\left\lfloor \left(O_{max}-O_{min}\right)\frac{fit_{i}-fit_{min}}{fit_{max}-fit_{min}}\right\rfloor +O_{min},$$

where $O_{i}$ represents the number of offspring of the *i*th chromosome, $O_{max}$ and $O_{min}$ are the maximum and minimum allowable numbers of breeding offspring, respectively, and $fit_{max}$ and $fit_{min}$ are the maximum and minimum fitness values in the formed population, respectively.

In addition, we proposed an adaptive regeneration strategy to improve the searching ability and robustness of the GATTRP. We recorded the elite chromosomes with the top fitness rankings in each round of iterations. When the elite chromosomes were not evolved within 20% of $I_{max}$ rounds in a row, the chromosomes in the bottom 10% of the genetic population were selected to perform a swap crossover with the elite chromosomes. By adopting an adaptive regeneration strategy to replace evolutionarily stagnant chromosomes, the diversity of the population was increased to improve the evolutionary ability of the algorithm.

### 4.4. Algorithm Flow

Based on the above theory, the pseudo-code of the GATTRP proposed in this paper is described in Algorithm 1.   
**Algorithm 1:** GATTRP.
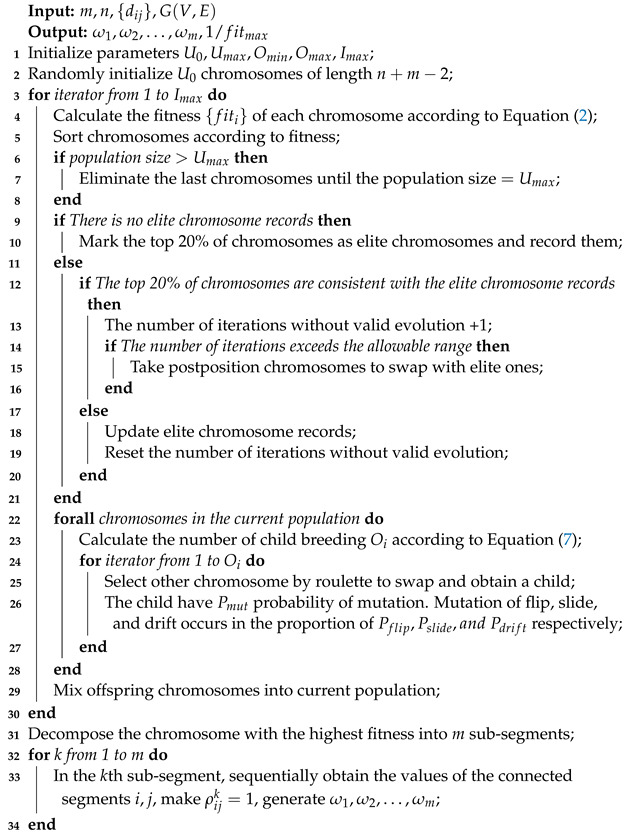



### 4.5. Working Pattern of GATTRP

We added a TT routing program based on GATTRP into the central control server of the smart factory TSN to optimize the TT routing. When a new TT subnetwork sent a registration message to apply to join the current TSN, the transmission continued normally while the GATTRP process was created to perform offline route planning. The newly joined subnetwork was treated as a node in *V* and the delay cost between it and other nodes was calculated to generate the new $\left\{d_{ij}\right\}$. The updated G(V,E) and $\left\{d_{ij}\right\}$ were input to the GATTRP process to calculate *m* new routes. Starting from the next time period, the central control server traversed the entire TT network with *m* new paths to complete the registration of the new subnetwork.

### 4.6. Convergence of GATTRP

GATTRP is based on a heuristic algorithm and the average time complexity of its convergence is complex and can be influenced by various factors, including population size, number of iterations, and randomness. Thus, it was difficult to produce an accurate expression. For its convergence, we could only estimate its time complexity as $O(U_{max}\times O_{max}\times I_{max})$.

### 4.7. Key Innovations and Contributions of GATTRP

To the best of our knowledge, GATTRP transforms, for the first time, the TT transmission problem in a smart factory TSN into an MTSP problem for optimization, which is the key innovation of this paper in terms of the TT routing problem. In addition, in contrast to existing genetic algorithms, GATTRP adds an elite chromosome mechanism that alleviates the problem of the GA tending to fall into premature and difficult-to-search-for solutions. With this optimization, GATTRP has a higher probability of obtaining better results after the iterations.

## 5. Adversarial Ant Colony Optimization Algorithm for Solving Non-TT Routing Problem

Non-TT flows in TSNs are used for soft real-time or non-real-time applications, providing bounded worst-case end-to-end delay (WCD) but with a looser delay constraint than TT flows. Multiple types of data flows, including TT, AVB, and BE, are sent from multiple ESs, as well as presequential SWs, which are connected to ingress ports in a single SW. To solve the composite traffic scheduling problem, the IEEE 802.1 Qbv standard [49] defined the time-aware shaper (TAS) mechanism to achieve traffic scheduling for different priority queues in time windows by establishing gate control lists (GCLs). Under the condition of network clock synchronization, the GCL in TAS periodically controls the opening and closing of the egress gates of the corresponding priority queue. With the adoption of GCLs, the transmission rate can match the egress bandwidth while segregating traffic of different priority levels, thereby reducing the interference of low-priority traffic on high-priority traffic and avoiding the starvation of low-priority traffic as much as possible.

**Figure 7:**
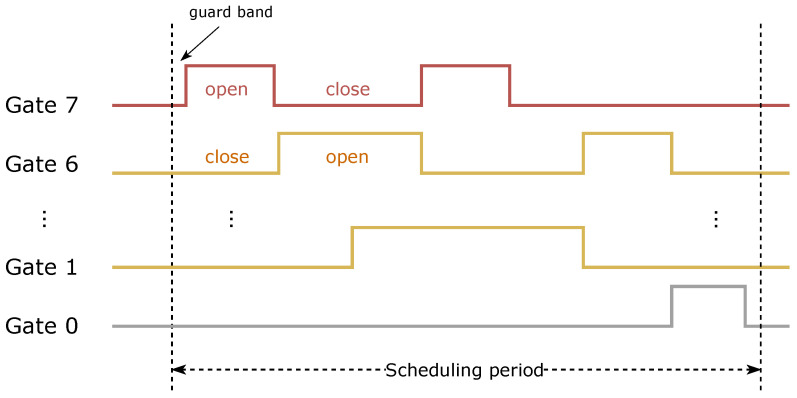
An example of TAS scheduling timing in a TSN (corresponds to Figure 8).

Typically, the SWs keep a total of eight priority queues, so the sequence of gates is Gate 7 to Gate 0. In the examples in this paper, “o” means gate open, “C” means gate closed, and each action is based on the time window. Correspondingly, the GCL can tell the time sequence of each type of traffic that is allowed to be sent to the output ports in a scheduling cycle, as shown in Figure 7.

In this scheduling mechanism, the number of time windows allocated to each priority queue within a common time period is limited. Therefore, as the number of queueing flows increases, it inevitably leads to relative congestion, which eventually causes an increase in the time needed to complete the transmission of each single task. For non-TT flows, ESs connected to each SW may send multiple types of non-TT data with different priorities, such as audio, video or sensor signals. The destination of non-TT flows is basically concentrated on one centralized data server. In this case, when the routing mechanism assigns too many time-consuming non-TT flows to the same data link, it is difficult to meet the soft deadlines of all flows due to severe time slot contention, resulting in a waste of bandwidth resources. When planning routes for low-priority non-TT flows, we considered changing their routing scheme from simply taking the shortest routing approach. When there were already higher priority flows causing queue congestion, we considered “bypassing” the congested SWs in exchange for a relative balance of SW loads at the cost of a partial loss of route length.

### 5.1. Definition of the Optimization Problem in Non-TT Routing

Based on the above conditions, the non-TT routing problem in TSNs could be defined as follows: in a given G(V,E) with *n* nodes, the planning of *m* routes for *m* non-TT flows with the sets of source nodes of these routes was defined as $SRC=\{v_{src_{1}},v_{src_{2}},\cdots ,v_{src_{m}}\}$, where the nodes are all repeatable. The destination of all non-TT flows was a centralized data server node $v_{dst}$. The priority of the *k*th non-TT flow was $p_{k}$, which was also repeatable, and the set of its packet lengths was $PCK=pck_{1},pck_{2},\cdots ,pck_{m}$. The route of the *k*th non-TT flow was denoted as $\omega_{k}=\left(v_{src_{k}},v_{2},v_{3},\cdots ,v_{dst}\right)$, which represented a path passing through *n* nodes of $v_{src_{k}}\to v_{2}\to v_{3}\to \cdots \to v_{dst}$.

We retained the description of the time delay from Section 4.1, with the difference that for this problem, the non-TT flow did not need to traverse each ES node inside the subnetworks, so the time delay of the *k*th non-TT flow between $v_{i},v_{j}$ nodes in the routes $\omega_{k}$ was:

(8) $$d_{ij}^{k}=c_{ij}+q_{ij}^{k},$$

where $q_{ij}^{k}$ is the queueing delay of the *k*th non-TT flow between $v_{i},v_{j}$. The higher the number of non-TT flows involved in queuing on any single SW node, the larger the WCD of the flows with a higher priority than the current *k*th flow, thereby making $q_{ij}^{k}$ increase correspondingly. The correspondence between the number of non-TT flows in a queue and $q_{ij}^{k}$ was determined by the GCL-based scheduling mechanism in the TSN, which was obtained through the simulation experiments detailed in later sections of this paper.

Since the traffic scheduling principle of a TSN requires planning higher priority non-TT flows first, the $\omega_{i}$ with the highest $p_{i}$ had to be prioritized, followed by planning the rest of flows in order of priority. The minimum value of the overall time delay *T* was:

(9) $$T=\sum_{k=1}^{m}\sum_{i=1}^{n}\sum_{j=1}^{n}d_{ij}\cdot \rho_{ij}^{k},$$

where $\rho_{ij}^{k}$ is the transmission direction of the non-TT frame between nodes $v_{i}$ and $v_{j}$, which was defined as follows:

(10) $$\rho_{ij}^{k}=\left\{\begin{array}{cc} 1, & \mathrm{non}-\mathrm{TT}\;\mathrm{frame}\;k\;\mathrm{goes}\;\mathrm{from}\;i\;\mathrm{to}\;j\\ 0, & \mathrm{non}-\mathrm{TT}\;\mathrm{frame}\;k\;\mathrm{goes}\;\mathrm{from}\;j\;\mathrm{to}\;i \end{array}\right..$$

In addition, we defined $y_{i}^{k}$ to mark whether the *k*th non-TT frame had visited the node $v_{i}$. Similarly, each node $v_{i}$ was marked one time per non-TT frame arrival; therefore, for every node $v_{i}$, there was:

(11) $$y_{i}^{k}=\sum_{j=0}^{n}\rho_{ji}^{k},\;\forall i\in \left\{1,2,\cdots ,n\right\},\;k\in \left\{1,2,\cdots ,m\right\}.$$

### 5.2. Optimization Goal and Constraints of Non-TT Routing

When non-TT flows with multiple priorities participate in queuing within the same SW, TAS schedules all priority queues in units of time windows. In a single SW, as the number of flows participating in the priority queue increases, the WCD of each data flow gradually increases, which is brought about by the deterioration of the queuing situation. The optimization goal of the AACO algorithm was to avoid unnecessary queuing as much as possible and, therefore, reduce the overall WCD for all flows, realize the relative balance of the TSN data link loads, and improve the response speed and bandwidth utilization of the non-TT networks, namely:

(12) $$\begin{array}{ccc} \; & \; & Minimize\{T\},\\ s.t. & (c1): & T_{i}<T_{j},\forall p_{i}>p_{j}\\ \; & (c2): & y_{i}^{k}\leq 1,\;\forall i\neq dst,k\in \left\{1,2,\cdots ,m\right\},\\ \; & (c3): & \sum_{k=1}^{n}y_{dst}^{k}=m,\\ \; & (c4): & T\leq MaxTime. \end{array}$$

In Equation (12), (c1)–(c4) are the constraints that needed to be obeyed by any solution. Constraint (c1) ensured that non-TT flows with higher priority always reached the destination earlier, where $T_{i}$ and $T_{j}$ are time delay for two different routes $\omega_{i}$ and $\omega_{j}$. Constraint (c2) ensured that every node $v_{i}$, except for $v_{dst}$, was visited by the *k*th non-TT flow no more than one time, so that there were no loops in the routes. Constraint (c3) ensured that the *m* non-TT flows all converged at the destination node $v_{dst}$. Constraint (c4) ensured that the final result *T* was less than the preset threshold MaxTime, otherwise its soft real-time performance was unsatisfied.

### 5.3. Description of AACO

**Figure 9:**
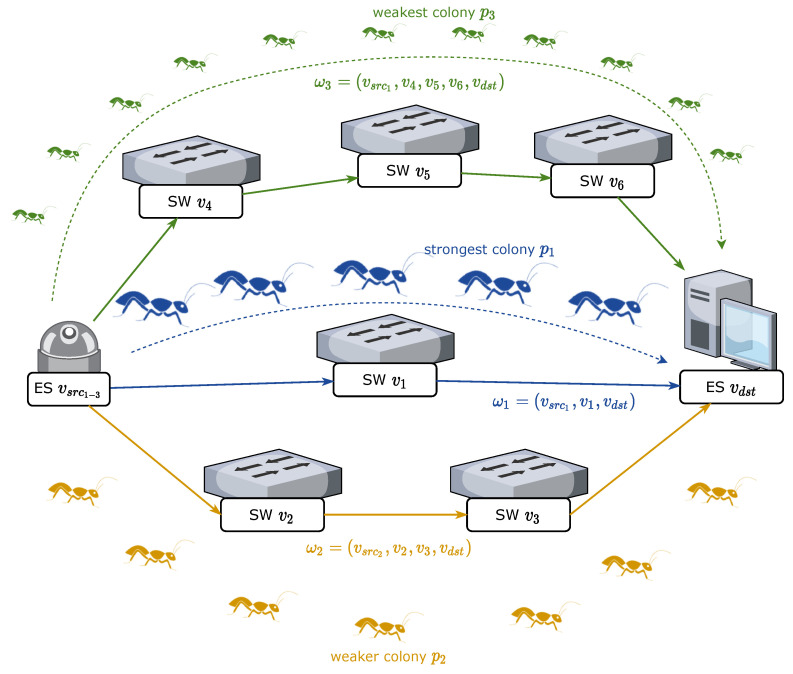
A sample procedure of the AACO algorithm solving the non-TT route assignment problem.

To solve the problem described above, we proposed a novel AACO algorithm to achieve load balancing routing among multi-priority non-TT flows. We set up *m* ant colonies, each of which represented a non-TT flow. Unlike classical ACO algorithms, there were strong and weak colonies among these ant colonies and the strength of the *i*th ant colony was equal to the priority $p_{i}$ of the *i*th non-TT flow. When the pheromone was updated, the pheromone left by the ant colonies with higher priorities greatly suppressed the pheromone increment of the weak colonies and reduced the probability of picking identical routes. Weak ant colonies would rather choose detouring than repeating the route of stronger colonies. Meanwhile, the distance to be detoured was also taken into account. When the extra cost of detouring was too large, weak ant colonies would choose to participate in the queuing process with stronger ant colonies, after weighing up the costs. Taking a small network consisting of six SWs with m=3 as an example, a sample procedure of the AACO algorithm solving the non-TT route assignment problem is shown in Figure 9.

#### 5.3.1. Ant Colony Initialization

For the route assigning task of *m* non-TT flows, we initialized *m* ant colonies with *Z* ants per colony. All ants from the *i*th colony were located at the corresponding source node $src_{i}$. We also initialized *m* different pheromone concentration values for each edge in *E*, denoted as $\tau_{ij}^{k}$:

(13) $$\tau_{ij}^{k}=C_{0},\forall i,j\in \left\{1,\cdots ,n\right\},\forall k\in \left\{1,\cdots ,m\right\}.$$

#### 5.3.2. State Transition Probability

We then calculated the state transition probability of the ants and selected the next node to visit by roulette, based on the state transition probability. The state transition probability was calculated as follows:

(14) $$P_{ij}^{kl}\left(t\right)=\left\{\begin{array}{cc} \frac{{\left[\tau_{ij}^{k}\left(t\right)\right]}^{\alpha }{\left[\eta_{ij}\left(t\right)\right]}^{\beta }}{\sum_{s\in allowed_{l}}{\left[\tau_{is}^{k}\left(t\right)\right]}^{\alpha }{\left[\eta_{is}\left(t\right)\right]}^{\beta }}, & j\in allowed_{l}\\ 0, & \mathrm{otherwise}. \end{array}\right.,$$

where $P_{ij}^{k}\left(t\right)$ is the probability that the *l*th ant in the *k*th ant colony chooses to visit $v_{j}$ from the current node $v_{i}$ at the time *t*, $allowed_{l}$ is the set of nodes that are directly accessible and have not been visited yet, $\tau_{ij}^{k}\left(t\right)$ is the pheromone concentration left on the edge $e_{ij}$ by the *k*th ant colony, and α is the pheromone influence factor and β is the cost function influence factor, both of which are preset constants. The cost function was defined as:

(15) $$\eta_{ij}\left(t\right)=\frac{1}{c_{ij}+\delta h_{i}},$$

where $h_{i}$ is SW hops between $v_{i}$ and $v_{j}$.

#### 5.3.3. Pheromone Update Rules

In each time window, the pheromone $\tau_{ij}^{k}\left(t\right)$ was volatilized in a fixed ratio. At the same time, the pheromone concentration was increased on the edges, according to the route traveled. For each ant in the *k*th colony:

(16) $$\tau_{ij}^{k}\left(t+1\right)=\left(1-\rho \right)\tau_{ij}^{k}\left(t\right)+\Delta \tau_{ij}^{k}\left(t\right),$$

where ρ is the volatility coefficient (preset between 0 and 1), $\Delta \tau_{ij}^{k}\left(t\right)$ is the sum of the pheromone increments in the *k*th population, which is accumulated by the pheromone increment of each ant $\Delta \tau_{ij}^{kl}\left(t\right)$, and *Q* is a preset pheromone increment constant. $\Delta \tau_{ij}^{kl}\left(t\right)$ was calculated by:

(17) $$\Delta \tau_{ij}^{kl}\left(t\right)=\left\{\begin{array}{cc} Q/c_{src_{k},dst}, & tour\left(i,j\right)\in tour\;\omega_{l}\left(v_{src_{k}},\cdots ,v_{dst}\right)\\ 0, & otherwise \end{array}\right..$$

Compared to existing ACO algorithms, the main improvement of the ACO in this paper was the calculation of pheromone increment $\Delta \tau_{ij}^{k}\left(t\right)$. The pheromone increment of each ant colony was calculated in decreasing order of priority. When calculating the *k*th pheromone increment, the pheromone increments of the previous k−1 ant colonies were taken into account. Ant colonies with higher priorities had a stronger suppression effect on the current colony. Therefore, when we calculated $\Delta \tau_{ij}^{k}\left(t\right)$, the sum of the pheromone increments of stronger ant colonies on the path to $v_{j}$ had a proportional negative impact on the current pheromone increment. $\Delta \tau_{ij}^{k}\left(t\right)$ was calculated by:

(18) $$\Delta \tau_{ij}^{k}\left(t\right)=\left\{\begin{array}{cc} \sum_{l=1}^{m}\Delta \tau_{ij}^{kl}\left(t\right)-\gamma \sum_{s=p_{k}+1}^{max(p)}\Delta \tau_{*j}^{s}\frac{s\cdot pck_{s}}{\sum_{h=1}^{max(p)}h\cdot pck_{h}}, & if>0\\ 0, & otherwise \end{array}\right.,$$

where γ is a preset constant whose purpose is to adjust the influence of stronger colonies on the current colony, $\Delta \tau_{*j}^{s}$ is the sum of pheromone increments on the edges of all paths whose priority is *s* and whose destination is $v_{j}$, and max(p) is the maximum value of priority *p*. The colonies with higher priorities than the *k*th colony were $\left(p_{k}+1,\cdots ,max\left(p\right)\right)$.

### 5.4. Algorithm Flow

Based on the above theory, the pseudo-code of the AACO algorithm proposed in this paper is described in Algorithm 2.
**Algorithm 2:** AACO algorithm for non-TT flow route planning.
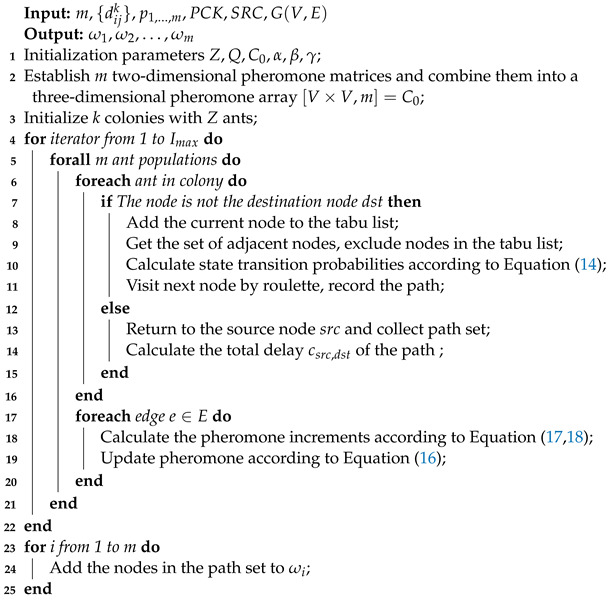



### 5.5. Working Pattern of AACO

We added a non-TT routing program based on the AACO into the central control server of the smart factory TSN to optimize non-TT routing. When a new non-TT application sent a registration message to apply to set up a data flow with the data server, the transmission continued normally while the AACO process was created to perform offline route planning. The ES node where the application was running was treated as the source node $v_{src_{k}}$ and the delay cost between it and other nodes was calculated to generate the new $\left\{d_{ij}^{k}\right\}$. The updated G(V,E), $\left\{d_{ij}^{k}\right\}$, and the priority of this data flow $p_{k}$ were input to the AACO process to calculate *m* new routes. Starting from the next time period, all non-TT flows were transmitted with *m* new routes to complete the registration of the new non-TT application.

### 5.6. Convergence of AACO

AACO is also based on a heuristic algorithm and the average time complexity of its convergence is complex and can be influenced by various factors, including the number of non-TT flows, number of ants, number of iterations, and randomness. Thus, it was difficult to produce an accurate expression. For its convergence, we could only estimate its time complexity as $O(m\times Z\times n\times I_{max})$.

### 5.7. Key Innovations and Contributions of AACO

For the non-TT routing problem in TSNs, one of the contributions of this paper is the selection of ACO as the basis of algorithm optimization. By reasonably adjusting the impact factor of the pheromones of stronger ant colonies on the update of the pheromones of other ant colonies, the improved ACO could effectively balance the relationship between the greedy principle and load balancing in order to better adapt to the TSN traffic shaping mechanisms, which is harder or even impossible for other algorithms to handle.

## 6. Performance Evaluation

In this section, we present simulation experiment environment that was used to evaluate the performances of the algorithms proposed in this paper when adopted in the routing scenarios of large-scale TSN networks in smart factories.

### 6.1. Single SW Traffic Scheduling Experiment Based on NeSTiNg

**Figure 10:**
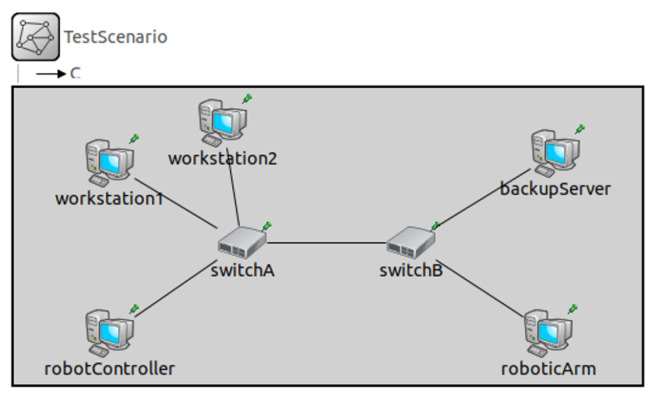
TSN topology of the simulation experiment that was built based on NeSTiNg.

Currently, there are two mainstream experimental frameworks in the field of TSN research, namely NeSTiNg [50] and CORE4INET [51]. NeSTiNg is an open-source project on the GitLab website [52], which was released in 2019 specifically for TSN simulation. Using the NeSTiNg simulation framework, Luxi Zhao et al. [53,54,55,56] accomplished a series of research works on network calculus-based TSN network latency analysis and optimization. In order to prepare the raw data on the time delay from a single SW for the routing algorithm proposed in this paper, we built a TSN simulation experimental environment based on NeSTiNg and set up the network topology depicted in Figure 10.

**Figure 11:**
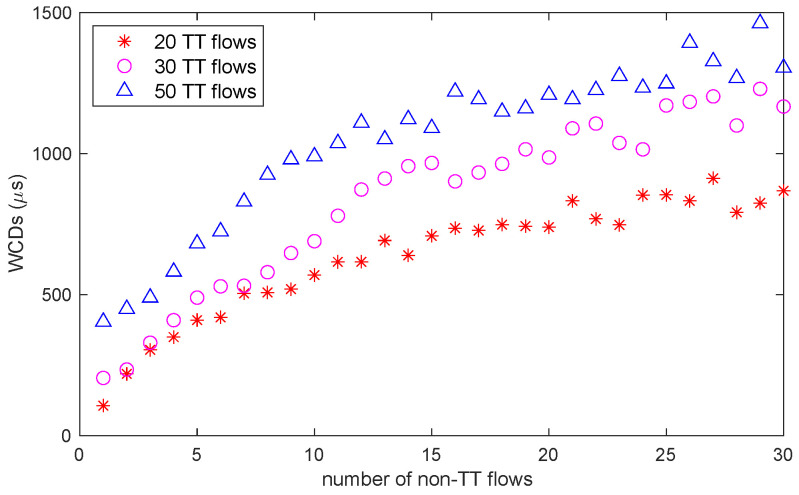
WCD changes with the quantity of flows participating in the queue.

On the experimental network, we deployed the end-to-end latency optimization methods proposed in [53,54]. For all flows that were transmitted through switchA, we changed the quantity of TT and non-TT flows participating in queuing and performed several sets of comparative experiments to quantify the correspondence between the WCD and queuing congestion, as depicted in Figure 11. Based on these data, we were able to calculate the correspondence between the queuing delay $q_{ij}^{k}$ and the quantity of flows to be transmitted to $v_{j}$ when the routes of non-TT flows overlapped, which was previously discussed in Section 5.1.

### 6.2. TT Flow Routing Experiment Based on GATTRP

**Figure 12:**
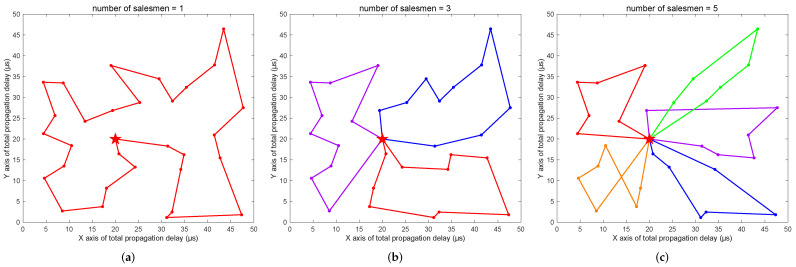
Comparison of traversal routes when the number of salesmen changed: (**a**) traversal route of one salesman; (**b**) traversal routes of three salesmen; (**c**) traversal routes of five salesmen.

In order to verify the effectiveness of the GATTRP proposed in this paper, we built a simulation experiment environment for the TT routing problem on the MATLAB® R2021b software platform and developed a test program based on Algorithm 1. The simulation program is available online at [57]. We simulated a factory with 30 TT subnetworks, in which each subnetwork was abstracted as a node. When visualizing the output results, the central control server was marked with a red pentagram, the traversal routes of *m* TT frames were represented by line segments, and different routes were labeled with different colors. Finally, on the longest route, all propagation and processing delays that were incurred while passing through each subnetwork were summed to calculate the final output *T*. The preset simulation parameters are shown in Table 2. We ran the experiments 30 times for each value of *m* and took the average value as the final result for each statistic in order to avoid misguidance by randomness. Figure 12a–c present some of the routing results obtained during the experiments.

**Figure 13:**
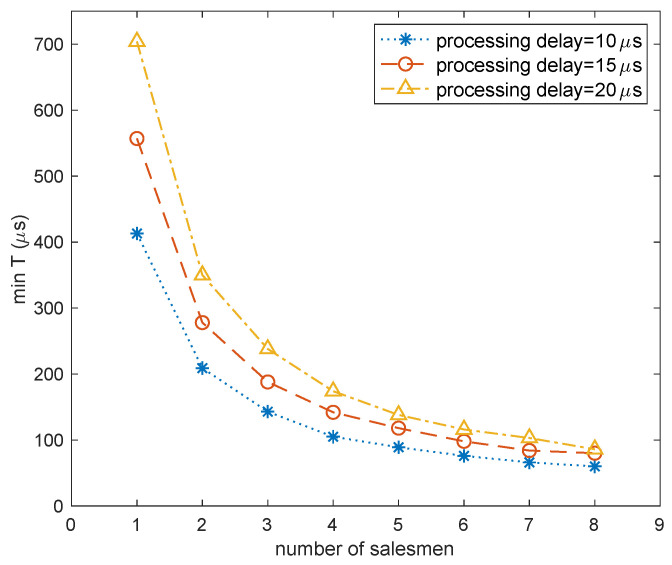
The effect of changing the number of salesmen on min(T) when using Algorithm 1.

As the TT flows traversed through each SW, they consumed a certain amount of processing delay. We set a random processing delay for each SW with maximum values of 10 μs, 15 μs, and 20 μs to simulate the change of the optimization target min(T) using Algorithm 1 when the number of salesmen *m* changed. The results are shown in Figure 13.

From the experimental results, we could confirm that it was feasible and effective to solve the routing problem of TT nodes based on the idea of solving the MTSP. With an increase in the number of salesmen, SW hops on the routes decreased and the propagation and processing delays on the longest route were correspondingly reduced. Considering that the number of parallel ingress and egress ports of the central control server is limited and that network wiring has comprehensive limitations from environment and financial costs, the value of *m* had to be properly set within a reasonable range. In addition, we observed that the higher the processing delay of the subnetworks, the better the optimization effect. Therefore, the larger the overall network size, the more obvious the optimization effect of the GATTRP.

**Figure 14:**
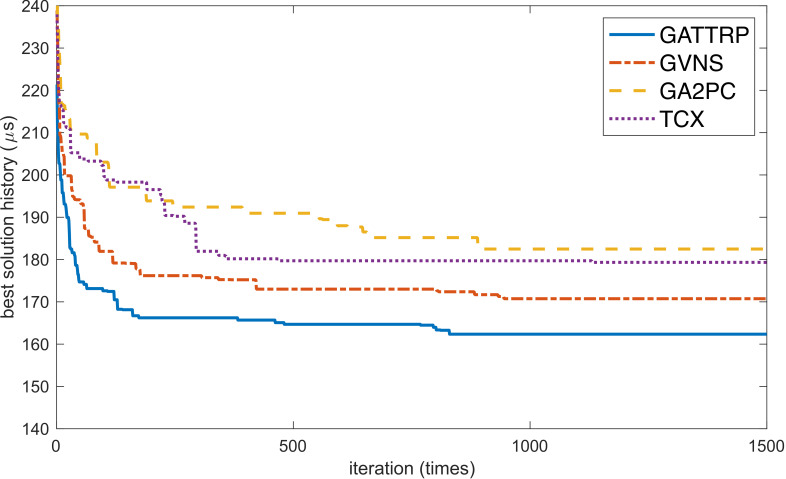
Convergence curves of the four algorithms when solving the same MTSP problem.

To illustrate the operational efficiency of Algorithm 1, we conducted comparative experiments using several other algorithms. Figure 14 shows GVNS, which is the general variable neighborhood search algorithm proposed by Soylu [58], GA2PC, which is the two-segment GA proposed by Carter et al. [34], TCX, which is the improved GA with a two-part chromosome crossover operator proposed by Yuan et al. [59], and GATTRP, which is Algorithm 1 from this paper. All four algorithms were tested using the same G(V,E) with m=5 and a processing delay of up to 20μs. The convergence curves are shown in Figure 14. The results showed that GATTRP converged faster and the final result after convergence was better.

### 6.3. Non-TT Flow Routing Experiment

In order to verify the effectiveness of Algorithm 2, as proposed in this paper, we developed a non-TT routing test program, which is available online at [57]. The simulation parameters are shown in Table 3. Likewise, we performed each experiment 30 times and took the average result as the final result to minimize misguidance caused by randomness.

**Figure 15:**
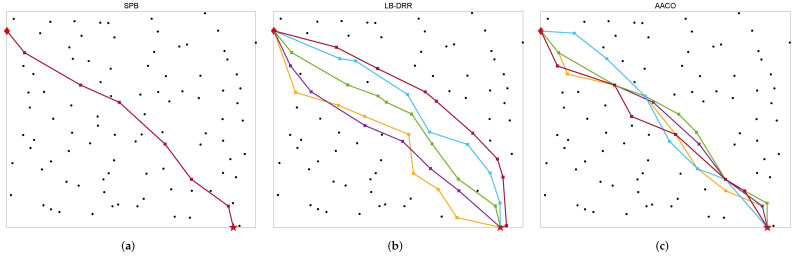
Comparison of the three algorithms for non-TT routing: (**a**) routes assigned by the SPB; (**b**) routes assigned by the LB-DRR; (**c**) routes assigned by the AACO.

We simulated a case of sending *m* non-TT flows from industrial sensors to a data server and compared the AACO to two other routing algorithms, as depicted in Figure 15. To make the visualization more intuitive, we chose the smaller *m* value of 5 and set the starting and end nodes as the two farthest nodes within the network. The starting node was labeled with a diamond and the end node with a pentagram. The route of each non-TT flow was represented by a line segment, which were distinguished from each other by color.

We developed a routing program based on the SPB algorithm proposed by [20] and the route obtained is depicted in Figure 15a. Since the routes planned by SPB for each non-TT flow are always the shortest solution, when the source node and the destination node were the same for all *m* flows, the obtained multiple paths completely coincided. In addition, we made appropriate modifications for our problem model based on the LB-DRR method proposed by Ojewale et al. [19]. The results of the LB-DRR algorithm are depicted in Figure 15b. For LB-DRR, the routes never overlapped with other routes when there were other options. In contrast, the route scheme generated by Algorithm 2 from this paper was more balanced and reasonable.

**Figure 16:**
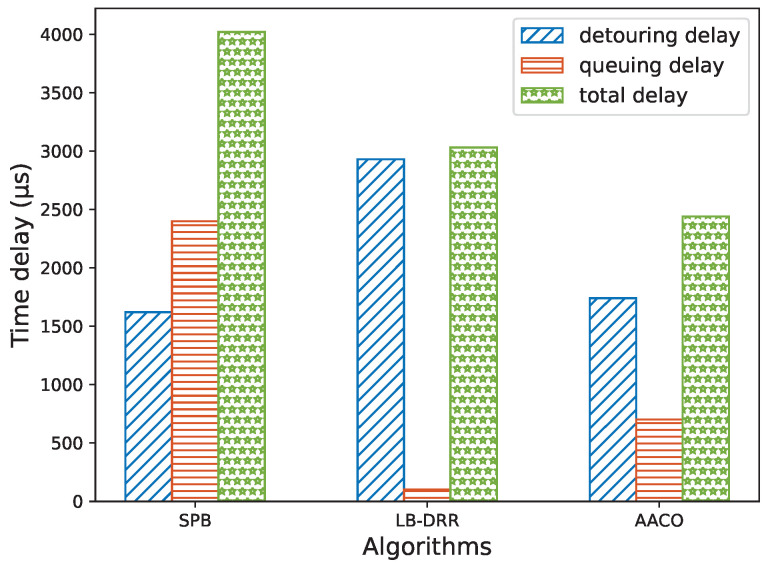
Comparison of the three algorithms when solving the same non-TT routing problem.

When a flow chose to detour, the more SWs the route passes through, the longer the processing and propagation delays. We defined the sum of processing and propagation delays as the detouring delay. In addition, from the simulation results presented in Section 6.1, we obtained the correspondence between queuing delay and the number of queuing flows, so we could estimate the queuing delay. We counted the average total delay from multiple experiments to evaluate the algorithm performances, as depicted in Figure 16. The results showed that the SPB, which pays attention to the shortest path, had the best detouring delay but the queuing delay was too long, resulting in the longest total delay. The LB-DRR, which focuses on load balancing, had the best queuing delay but due to too many detours, results were also unsatisfactory. The AACO algorithm proposed in this paper comprehensively considered a balanced distribution along with the optimization of routes; thus, the total delay was the shortest and the optimization effect was the best.

## 7. Conclusions

This paper highlighted the routing problems in TSNs. Different from traditional bus-based real-time communication networks, TSNs have two main routing problems: those for TT flows and those for non-TT flows. To address the specific communication needs of smart factories, we classified network data from smart factories and analyzed the communication requirements and transmission mechanisms of various types of traffic. By mathematically modeling the problem, we transformed the routing problem into an MTSP problem and a load-balanced multi-priority route assignment problem. For the MTSP problem, we proposed an improved algorithm named GATTRP. For the multi-priority route assignment problem, we proposed an improved algorithm named AACO. The simulation results showed that transforming the traversal problem of TT flows into an MTSP can effectively reduce the traversal cycle time and that the GATTRP proposed in this paper has a strong convergence performance. For non-TT flows, the AACO proposed in this paper is more comprehensive and exhibits an excellent performance and better results. However, there were also some limitations in this paper. For upper-layer industrial applications, the transmission mechanisms and requirements of AVB flows and BE flows, which belong to the same non-TT flow type, are not exactly the same. If they can be distinguished for refined routing, the the routing performance could be further improved. In the future, we will focus on improving the routing algorithms for AVB flows.

## Figures and Tables

**Figure 8:**
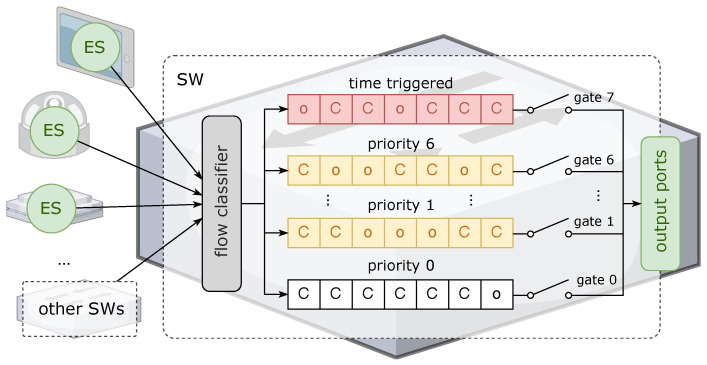
TAS scheduling mechanism with GCLs in a TSN.

**Table 1:** TSN data classification.

Category	Sample Data	Description	Priority
TT	Control Data Frames	Communicate with industrial control slave devices, such as servo motors, on a strictly time cycle basis to control their actions and collect encoder feedback	7
	Time-Synchronized Frames	Traverse each network node following the rules of the optimal master clock algorithm to complete precise time synchronization	
AVB	Audio Bridging Data	Sensing signals, such as vibration, sound, etc., typically requiring latency to be less than 5 ms	5∼6
	Video Bridging Data	Continuous image signals captured by industrial surveillance cameras with large bandwidth consumption: allowable time delay range is 0∼100 ms	
	Key Sensor Data	Event-triggered multi-source heterogeneous sensor signal data, which is an important data source for realizing intelligent manufacturing management	
BE	ERP, MES, etc. System Data	Generic Ethernet data with no particular real-time QoS requirements	0∼4
	Background Stream Data	Deliver as much as possible	

**Table 2:** TT flow routing simulation parameters.

Symbol	Value	Description	Remarks
*n*	30	The number of nodes to be traversed	Can be selected by the user
$U_{0}$	100	The size of the initial population	Can be selected by the user
$U_{max}$	150	The maximum size of the chromosome population	Can be selected by the user
$I_{max}$	1500	The maximum number of iterations	Parameter of the algorithm
$P_{mut}$	40%	The probability of mutation	Parameter of the algorithm
$P_{flip}$	25%	The probability of flip mutation	Parameter of the algorithm
$P_{slide}$	25%	The probability of slide mutation	Parameter of the algorithm
$P_{drift}$	50%	The probability of drift mutation	Parameter of the algorithm

**Table 3:** The non-TT routing simulation parameters.

Symbol	Value	Description	Remarks
*n*	100	The number of SWs in the non-TT network	Can be selected by the user
*Z*	80	The number of ants in each colony	Parameter of the algorithm
*Q*	2	The pheromone increment constant	Parameter of the algorithm
$C_{0}$	2	The initial pheromone constant	Parameter of the algorithm
$I_{max}$	800	The maximum number of iterations	Parameter of the algorithm
α	1	The pheromone impact factor	Parameter of the algorithm
β	5	The heuristic impact factor	Parameter of the algorithm
ρ	0.5	The pheromone volatile factor	Parameter of the algorithm
γ	0.05	The stronger ant colony impact factor	Parameter of the algorithm

## Data Availability

The data presented in this study are available upon request from the corresponding author. They are restricted to experimental results.
